# Selection rules of triboelectric materials for direct-current triboelectric nanogenerator

**DOI:** 10.1038/s41467-021-25046-z

**Published:** 2021-08-03

**Authors:** Zhihao Zhao, Linglin Zhou, Shaoxin Li, Di Liu, Yanhong Li, Yikui Gao, Yuebo Liu, Yejing Dai, Jie Wang, Zhong Lin Wang

**Affiliations:** 1 Beijing Institute of Nanoenergy and Nanosystems, Chinese Academy of Sciences, Beijing, P. R. China; 2 School of Materials, Sun Yat-sen University, Guangzhou, P. R. China; 3 School of Nanoscience and Technology, University of Chinese Academy of Sciences, Beijing, P. R. China; 4 School of Materials Science and Engineering, Georgia Institute of Technology, Atlanta, GA USA

**Keywords:** Energy, Devices for energy harvesting, Materials for devices

## Abstract

The rapid development of Internet of Things and artificial intelligence brings increasing attention on the harvesting of distributed energy by using triboelectric nanogenerator (TENG), especially the direct current TENG (DC-TENG). It is essential to select appropriate triboelectric materials for obtaining a high performance TENG. In this work, we provide a set of rules for selecting the triboelectric materials for DC-TENG based on several basic parameters, including surface charge density, friction coefficient, polarization, utilization rate of charges, and stability. On the basis of the selection rules, polyvinyl chloride, used widely in industry rather than in TENG, is selected as the triboelectric layer. Its effective charge density can reach up to ~8.80 mC m^−2^ in a microstructure-designed DC-TENG, which is a new record for all kinds of TENGs. This work can offer a basic guideline for the triboelectric materials selection and promote the practical applications of DC-TENG.

## Abstract (web summary)

Appropriate triboelectric material selection is vital to for high performance direct current triboelectric nanogenerator (DC-TENG). The authors here provide effective selection rules as guideline to select triboelectric materials for DC-TENG to reduce the trial-and-error cost for DC-TENG’s research.

## Introduction

With the development of the Internet of Things (IoTs) and artificial intelligence (AI), our daily life is embracing unimaginable and complex distributed arrays of electronics and sensors, which drive urgent demand for distributed energy harvesters^1–3^. Triboelectric nanogenerator (TENG), as the energy supply units and the self-powered sensor units, represents one of the core groups of potential technologies with great potential applications to support the fast development of IoTs and AI^4–7^. In addition, its high efficiency at low frequency, light-weight, and low cost make it a prime candidate for integration with multifunctional electronics^8–13^. According to the working mechanism and output signal type, TENG can be divided into alternative current TENG (AC-TENG) based on contact electrification and electrostatic induction effects, and direct-current TENG (DC-TENG) based on contact electrification and electrostatic breakdown effects. Among them, DC-TENG possesses more advantages, such as driving electronics directly without a rectifier unit, anti-electromagnetic interference of output signal, and no limitation of dielectric breakdown^14–17^.

The excellent output performance of TENG, namely high effective charge density, is the essential prerequisite as an energy harvester or a self-powered sensor^18–21^. Very recently, the limiting factor of charge density of DC-TENG (*σ*_DC-TENG_) has been described as^13^:

1 $${\sigma }_{{{{{{\rm{DC}}}}}}-{{{{{\rm{TENG}}}}}}}=k\,\times \,{{{{{\rm{min}}}}}}({\sigma}_{{{{{{\rm{triboelectrification}}}}}}},{\sigma}_{{{{{{\rm{c}}}}}},\;{{{{{\rm{electrostatic}}}}}}\;{{{{{\rm{breakdown}}}}}}})$$

where *k* is the electrode structure factor, *σ*_triboelectrification_ is the triboelectrification charge density, and *σ*_c, electrostatic breakdown_ is the collected effective charge density by the electrostatic breakdown. The *k* and *σ*_c, electrostatic breakdown_ have been optimized to significantly enhance the output of DC-TENG by electrode structure design or external environment controlling (e.g., temperature or atmosphere), and the effective charge density has reached a new milestone of 5.4 mC m^−2^^12,15,22^. The *σ*_triboelectrification_ is the basic parameter for contact electrification effect, which largely determines whether the electrostatic breakdown (generally is air breakdown for DC-TENG in the air) occurs or not^15^, and has a close relationship with the species of triboelectric materials. Thus, the triboelectric materials series has been provided as a guideline for the materials selection of TENG^23^, however, it is only applicable for the AC-TENG, especially for contact-separate AC-TENG. As for the DC-TENG, a kind of sliding TENG derived from the electrostatic breakdown, except for the *σ*_triboelectrification_, more complex parameters (friction properties, dielectric properties, etc.) should be considered in order to select suitable triboelectric materials. Thus, providing a guideline for selecting appropriate triboelectric materials is the key to designing a high-performance DC-TENG and promoting the practical applications of DC-TENG.

In this work, we propose a set of selection rules to determine whether a kind of triboelectric material is suitable for DC-TENG. Taking the surface charge density, friction coefficient, strength of polarization, the utilization rate of triboelectric charges, and stability as basic parameters, we find that the triboelectric material of polyvinyl chloride (PVC) film, which is widely utilized in the industry but not in TENG field, showed an excellent DC output performance. The effective charge density for a microstructure-designed DC-TENG with PVC can reach ~8.80 mC m^−2^, setting a new TENG’s record. The proposed comprehensive model for selecting triboelectric materials not only can be used for DC-TENG to achieve high-performance DC-TENG, but also can provide a guideline for the applications of DC-TENG in practice.

## Results and discussion

### Judgement rules of triboelectric materials in DC-TENG

**Fig. 1:**
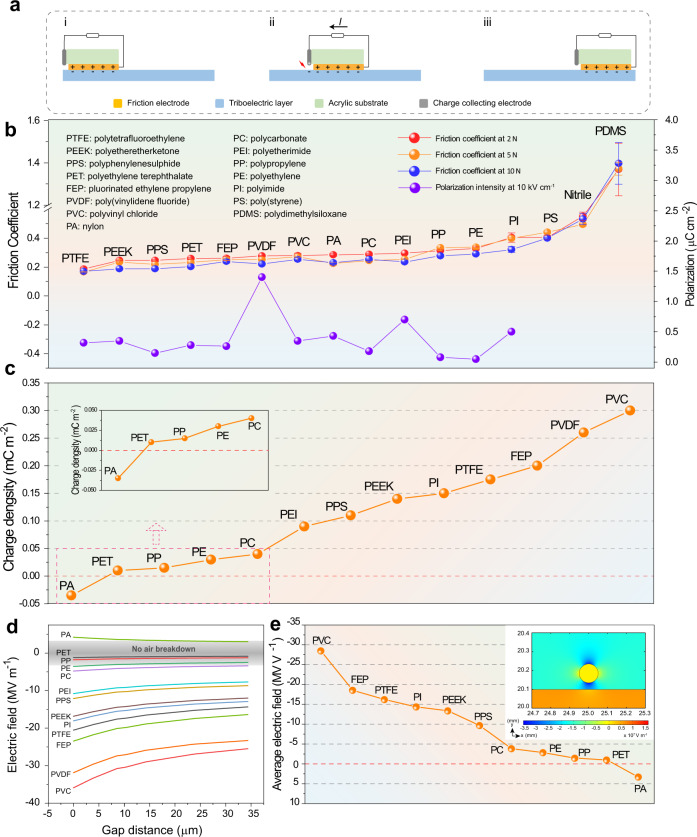
Friction and triboelectric performance of TENG with different triboelectric materials. **a** Schematic diagram of DC-TENG. **b** The friction coefficient (*μ*) between Cu with various triboelectric layers and the polarization intensity of triboelectric layers at 10 kV cm^−1^ (error bars represent standard deviation). **c** The surface charge density of different triboelectric layers. **d** The electric field distribution curves in the gap between CCE and triboelectric layers’ surface (simulated by COMSOL software) and **e** corresponding summary of average electric field (the inset is the simulated result of PVC film).

The working mechanism of DC-TENG is shown in Fig. 1a, which couples the contact electrification and electrostatic breakdown effects (detailed explanation is shown in Supplementary Note 1). The movement of DC-TENG is a kind of sliding process, and therefore the low friction coefficient (*μ*) is essential to obtain a high-efficiency DC-TENG. Here, taking generally used copper as the friction electrode (FE), the *μ* of various commercial polymers under different loads are tested (Fig. 1b). The test diagram is shown in Supplementary Fig. 1 and the calculation of *μ* is shown in Supplementary Note 2. The average *μ* of most dielectric films is <0.4 (Supplementary Fig. 2), and the polytetrafluoroethylene (PTFE) film and polydimethylsiloxane (PDMS) film presents the smallest *μ* of ~0.17 and the largest *μ* of ~1.35, respectively. The surface roughness *Ra* of the triboelectric materials has no obvious influence on their friction coefficient (Supplementary Fig. 3). The *Ra* value of PTFE is ~0.15 μm, which is larger than that of most of the triboelectric materials used in this work, but the *μ* of PTFE is the smallest. Large *μ* between the copper electrode and polymer film will increase the wear process during the sliding movement. Thus, the films with *μ* lower than 0.4 are utilized as the triboelectric materials to confirm their surface charge density (*σ*_SCD_) during the triboelectrification process. Consequently, the PDMS, Nitrile rubber, and poly(styrene) (PS) films are left out, although PDMS has been used as good triboelectric material in contact-separation type AC-TENG.

The sliding AC-TENG is utilized to confirm the *σ*_SCD_ between Cu and different triboelectric layers, as shown in Supplementary Fig. 4, and the detailed working mechanism is determined in Supplementary Note 3. The *σ*_SCD_ and short circuit current (*I*_sc_) is presented in Fig. 1c and Supplementary Fig. 5 (the slider of AC-TENG is 10 mm × 20 mm). The polyethylene terephthalate (PET), polypropylene (PP), polycarbonate (PC), and polyethylene (PE) show quite inferior *σ*_SCD_, which is <0.05 mC m^−2^. A moderate *σ*_SCD_ (0.05 mC m^−2^<*σ*_SCD_<0.15 mC m^−2^) is obtained when using polyetherimide (PEI), polyphenylenesulphide (PPS), polyimide (PI), or polyetheretherketone (PEEK) as the triboelectric layer. The rest films, e.g., PTFE, fluorinated ethylene propylene (FEP), poly(vinylidene fluoride) (PVDF), and PVC, provide large *σ*_SCD_, exceeding 0.15 mC m^−2^. Especially, the *σ*_SCD_ of 0.30 mC m^−2^ can be obtained when using PVC as the triboelectric material. For the sliding AC-TENG with nylon (polyamide, PA) film as triboelectric material, the flow direction of output charges is opposite to the other films, which indicates that PA film loses electrons during the contact electrification process with Cu electrode.

According to the working mechanism of DC-TENG, the electrostatic breakdown occurs in the gap between triboelectric film and charge collecting electrode (CCE) due to the electrostatic field generated by the charges on the surface of triboelectric layers (Supplementary Note 1). The electric field distribution in the gap is simulated by COMSOL software and provided to depict the intensity difference of various triboelectric materials (from a to b point in Supplementary Fig. 6), where the *σ*_SCD_ is set according to the results in Fig. 1c. As shown in Fig. 1d, e and Supplementary Fig. 7, the electric field strength in the gap shows the same trend as their *σ*_SCD_. The whole/partial simulated electric curves of PA, PET, PP, PE, and PC films are in the non-breakdown area (less than the air breakdown strength of 3 MV m^−1^). Thus, they only have a weak electrostatic field for air breakdown and generate a weak DC output for the DC-TENG or sometimes they even cannot cause air breakdown (e.g., PET and PP). It is worth noting that the electric field in the gap between PA and CCE is opposite to that of the other triboelectric films, resulting in the opposite DC signal in the external circuit. With the *σ*_SCD_ increasing, the electric field in the gap becomes stronger, which is beneficial for the air breakdown process, thereby increasing the number of charges collected by the CCE and improving the DC output performance^13^. Moreover, on the basic of triboelectrification effect and the mechanism of DC-TENG, the triboelectric charges not only generate the electrostatic field in the gap to achieve the air breakdown but also generate an electric field in the polymer near the surface (Supplementary Fig. 6), which might induce the internal polarization effect near the surface along the electric field and constrain part of surface charges, making them hard to participate in the air breakdown process (Supplementary Fig. 8). The polarization intensity (*P*) vs. electric field loops of different triboelectric materials except for the PS, Nitrile, and PDMS films were carried out because of their overlarge friction coefficient, as shown in Fig. 1b and Supplementary Figs. 9, 10. Compared with other polymer films, PVDF and PEI exhibit a stronger polarization effect at the same electric field, indicating that they show a stronger polarization effect under the same electric field. The large polarization means more dipolar under the electric field (Supplementary Fig. 8), meaning that more charges will be bound and the air breakdown process might be restrained in the PVDF and PEI film.

### Primary material selection rules of DC-TENG

**Fig. 2:**
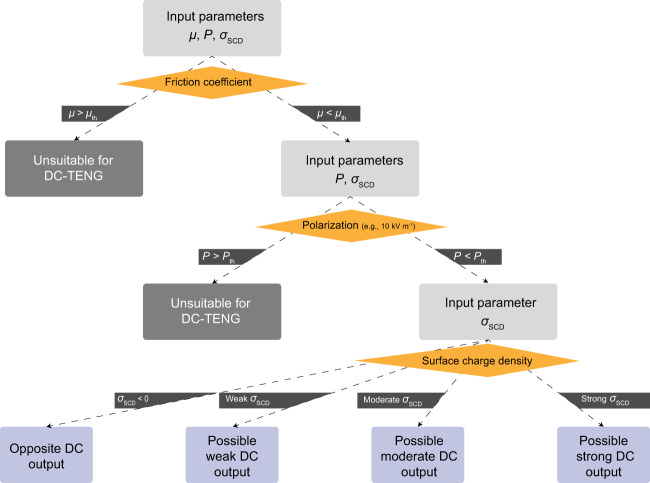
Primary selection rules of triboelectric materials for DC-TENG. The input parameters *μ* is friction coefficient, *P* is polarization, and *σ*_SCD_ is surface charge density.

According to the working mechanism of DC-TENG, based on the intrinsic parameters of *μ*, *P*, and *σ*_SCD_, as shown in Fig. 2, we can provide a model to formulate the primary selection rules, which can determine whether a material is suitable for use as the triboelectric material in DC-TENG, and also determine the possible DC output performance. As shown in Fig. 2, the primary selection rules can be divided into three steps. As shown in Fig. 2, first, the DC-TENG is a kind of sliding-mode TENG, which is based on the sliding friction process and triboelectrification between the electrode and film. Thus, an appropriate friction coefficient of the triboelectric film is needed to reduce the wear and heat during the friction process between the electrode and triboelectric film. Therefore, the triboelectric layer with overlarge *μ* should be excluded, and the threshold value *μ*_th_ is set as (~0.40), which is just a preliminary estimate for this primary rule based on the various triboelectric materials in this work. The *μ*_th_ in this work is the middle value between the average *μ* of Cu and PI (~0.38, Supplementary Fig. 2) and the average *μ* of Cu and PS (~0.42, Supplementary Fig. 2). Second, on the basis of the mechanism of DC-TENG, the strong polarization of the triboelectric layer has an adverse effect on the air breakdown process and decreases the DC output of DC-TENG. Thus, the triboelectric layer with overlarge *P* should be ruled out. Here, except for the PVDF and PEI, the polarization intensity of the rest triboelectric materials is always higher than 0.05 μC m^−2^ (for PE) but lower than 0.5 μC m^−2^ (for PI) at 10 kV m^−1^, as shown in Fig. 1b and Supplementary Figs. 9, 10. Therefore, the upper limit value of *P* (*P*_th_) is set as a preliminary estimate for this primary rule in this work, which is the middle value (~0.6 μC m^−2^ at 10 kV m^−1^) between 0.5 μC m^−2^ (polarization intensity of PI at 10 kV m^−1^) and 0.7 μC m^−2^ (polarization intensity of PEI at 10 kV m^−1^). It should be noted that the thresholds (*μ*_th_ and *P*_th_) are preliminary estimates based on the triboelectric materials used in this experiment and will be further refined as more materials are studied in future works. Third, the possible DC output of the remaining triboelectric materials can be estimated based on their respective *σ*_SCD_ value, and only the triboelectric materials with high *σ*_SCD_ can form a high DC output owing to the high electric field in the gap (Fig. 1d, e). These primary selection rules will provide a guideline to select appropriate triboelectric materials, and reduce the trial-and-error cost for DC-TENG’s research.

### Validation of material selection rules of DC-TENG

**Fig. 3:**
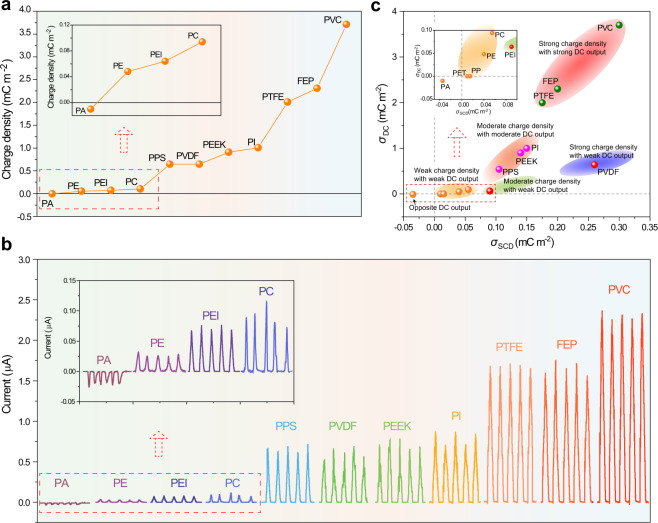
DC output performance and corresponding relationship with surface charge density. **a** Effective charge density and **b** short circuit current of microstructure-designed DC-TENG (DC unit: 20) with different triboelectric layers. **c** The relationship between surface charge density and effective charge density of DC-TENG of different triboelectric layers.

The DC output performance of these 13 kinds of triboelectric materials is determined by the microstructure-designed DC-TENG device (Supplementary Fig. 11a), whose size is similar to that of an AC-TENG device: 10 mm × 20 mm and DC unit = 20. The detailed structure is shown in Supplementary Fig. 11b. The interlaced FEs and CCEs are arranged on the acrylic substrate orderly, and a tiny air breakdown gap (30–40 μm) is formed between CCE and triboelectric layer (Supplementary Fig. 11c). The friction coefficients with a micro-structured copper electrode of various triboelectric materials are shown in Supplementary Fig. 12. The friction coefficients with a micro-structured copper electrode show a similar tendency with the friction coefficients with flat copper electrodes for the different triboelectric materials (Fig. 1b). The working mechanism is shown in Supplementary Fig. 13 and Note 4. As shown in Fig. 3a, b and Supplementary Fig. 14, the PET and PP do not have the DC output owing to their low *σ*_SCD_ value (Fig. 1d), and the PE, PEI, and PC present low DC effective charge density (*σ*_DC_) of <0.5 mC m^−2^ (*I*_sc_<0.15 μA), whereas the PI, PEEK, PPS and PVDF show moderate *σ*_DC_ in the range from 0.5 to 2.0 mC m^−2^ and corresponding *I*_sc_ of ~0.5 μA. The PVC, FEP, and PTFE provide high *σ*_DC_ and *I*_sc_, which is >2.0 mC m^−2^ and 1.5 μA, respectively. It should be noticed that, when the PA film is utilized as triboelectric material, the DC output is also opposite to the other friction films (Fig. 3b), because its surface charges are positive when in contact with Cu electrode. The working mechanism is shown in Supplementary Fig. 15 and Supplementary Note 5.

The relationship between *σ*_SCD_ and *σ*_DC_ of different triboelectric films is shown in Fig. 3c. It can be clearly seen that the PVDF and PEI films show relatively high surface charge density but low DC output, and moderate surface charge density but low DC output, respectively, indicating that they are not suitable as the triboelectric layers for DC-TENG due to their relatively strong polarization effect. Except for them, the DC output of the other triboelectric materials is increased with the increase of *σ*_SCD_. Meanwhile, the relationships between *σ*_SCD_ and *σ*_DC_ can be divided into four types: a. the films show strong surface charge density with strong DC output (e.g., PVC, FEP, PTFE); b. the films present moderate surface charge density and moderate DC output (e.g., PI, PEEK, PPS); c. the films show low surface charge density and low DC output (e.g., PC, PE, PP, PET); d. the film shows opposite DC output (PA). On one hand, the larger *σ*_SCD_ of triboelectric films will lead to a stronger electric field in the gap which is beneficial to the air breakdown effect. On the other hand, when the triboelectric film has a larger *σ*_SCD_, more surface charges on the film will participate in the air breakdown process and can be collected by DC-TENG. In other words, the utilization rate of contact electrification charges in single DC unit (*η*, the ratio of collected charges by one DC unit and the *σ*_SCD_) will increase. For example, the PVC, FEP, and PTFE films show high *η* of >60%, but the PE just has a low *η*, <10% (Supplementary Fig. 16). Therefore, this relationship between *σ*_SCD_ and *σ*_DC_ for different triboelectric films confirms the effectiveness of the selection rules in Fig. 2.

### Comprehensive material selection model of DC-TENG

**Fig. 4:**
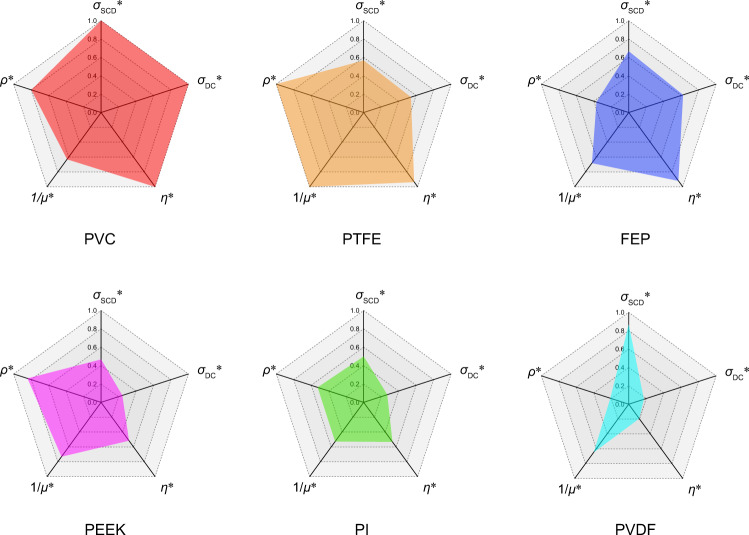
Comprehensive selection rules of triboelectric materials for DC-TENG. The radar chart of normalized indexes of PVC, FEP, PTFE, PEEK, PI, and PVDF, where the *σ*_SCD_*, *σ*_DC_*, η*, 1/μ*, and ρ* is the normalization of *σ*_SCD_: surface charge density, *σ*_DC_: DC charge density, *η*: (σ_DC_/20)/*σ*_SCD_, *1/μ*: the reciprocal of friction coefficient, *ρ*: stability, respectively.

In practical applications, one or more key indexes should be decided to satisfy the application requirements of DC-TENG usage. For example, for energy harvester, the triboelectric materials should possess a high DC output and high *η*. For sensors, the stability of triboelectric materials with an appropriate voltage/current output is necessary to maintain repeatability and accuracy. Therefore, in the radar chart shown in Fig. 4, a comprehensive model of the performance selection of triboelectric materials for DC-TENG is given. Five parameters are shown in Supplementary Table 1, *σ*_SCD_*, *σ*_DC_*, η*, 1/μ*, and ρ*, is the normalization of *σ*_SCD_, *σ*_DC_, *η*, the reciprocal of the friction coefficient (1/*μ*), and the stability (*ρ*, shown in Supplementary Fig. 17 and Note 6), respectively. They are utilized as the performance evaluation indexes of triboelectric materials in DC-TENG (taking PVC, FEP, PTFE, PEEK, PVDF, and PI as the representative materials), as shown in Fig. 4. The PVC, FEP, and PTFE films present high *σ*_SCD_ values as well as high DC output. At the same time, they also provide a high utilization rate of contact electrification charges in the air breakdown process, with *η* reaching ~60% (Supplementary Table 1). However, FEP film shows inferior stability for DC-TENG because its surface layer is easily stripped off during the stability test (Supplementary Fig. 18), which will jam the gap and make the air breakdown hard to occur (Supplementary Fig. 19). Both of the PVC and PTFE films have outstanding comprehensive properties, and the PVC shows a larger effective charge density and PTFE shows a better friction property. Compared with the PVC, FEP, and PTFE, the PEEK and PI film show relatively moderate comprehensive properties. However, the PVDF film has inferior properties with quite low *η* and poor stability, so it is not suitable for use as a triboelectric layer in DC-TENG.

### Application of selected PVC film for DC-TENG

**Fig. 5:**
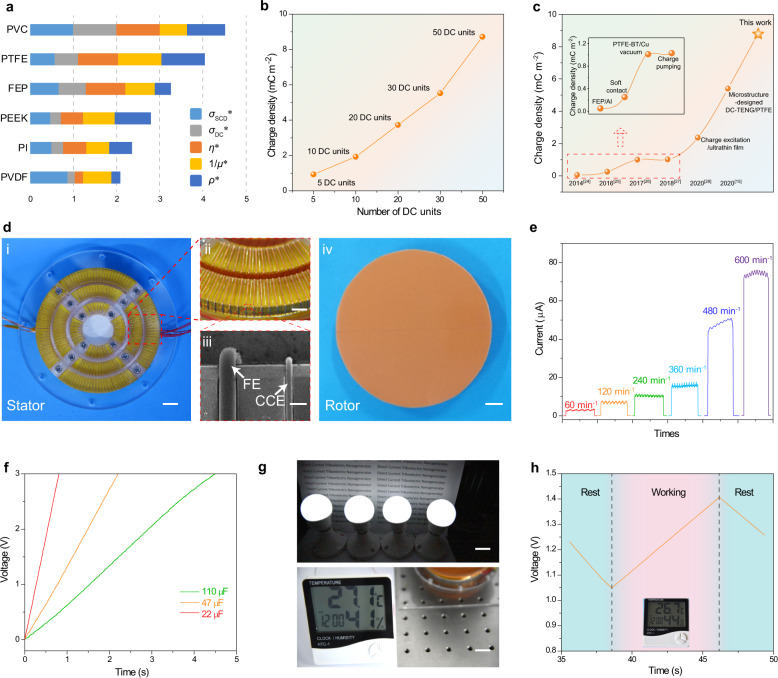
Application of selected PVC film as friction for DC-TENG. **a** The stacked bar plot of normalized properties of PVC, PTFE, FEP, PEEK, PI, and PVDF. **b** The effective charge density of microstructure-designed DC-TENG with different DC units. **c** Comparison of the charge density of TENGs^15, 24–28^. **d** Photograph of rotary DC-TENG ((i) stator (scale bar: 1.0 cm), (ii) partial enlarged detail of stator (scale bar: 1.5 mm), (iii) SEM image of stator (scale bar: 250 μm) and (iv) rotor (scale bar: 1.0 cm)). **e** Output current of rotary DC-TENG with PVC as triboelectric material under various rotating speeds. **f** Charging curves of capacitors (10, 47, 110 µF) charged by rotary DC-TENG with PVC as triboelectric material (600 r min^−1^). **g** Photograph of driving commercial LED bulbs and thermo-hygrometer directly by rotary DC-TENG with PVC as triboelectric material (600 r min^−1^, scale bar: 2.0 cm). **h** Monitored voltage of the 660  µF commercial capacitors with driving thermo-hygrometer simultaneously by rotating DC-TENG with PVC as triboelectric material (600 r min^−1^).

It can be seen that the PVC film shows good comprehensive properties as triboelectric material for DC-TENG owing to its largest stacked normalized indexes, as shown in Fig. 5a. Utilizing PVC as the triboelectric layer, DC-TENG can provide an increased effective charge density and current as the number of DC units increases: 0.82 mC m^−2^ and 0.56 μA for DC-TENG with 5 DC units, 8.80 mC m^−2^ and 5.88 μA for the DC-TENG with 50 DC units (sliding distance: 5 cm, sliding width: 1 cm, Fig. 5b and Supplementary Fig. 20). This highly effective charge density (8.80 mC m^−2^) is >20 times that of the previous DC-TENG^12^, and breaks the existing records of output charge density for various-type TENG (Fig. 5c)^15,24–28^.

The microstructure-designed DC-TENG was fabricated into rotary DC-TENG to realize the continuous DC output, whose device structure and microscopic photo are shown in Fig. 5d(i–iii). As the same structure with the sliding DC-TENG in Supplementary Fig. 13, the interlaced FEs and CCEs are orderly arranged on the surface of the acrylic substrate (Fig. 5d(i, ii)), and the distance between FE and adjacent CCE is ~750 μm (Fig. 5d(iii)). The PVC film is attached to the sponge, which is utilized as the rotor (Fig. 5d(iv)). With the rotating speed gradually increasing, the continuous DC output of rotary DC-TENG also increases from 3.3 μA to 75 μA (Fig. 5e). Utilizing the 22 μF, 47 μF, and 110 μF capacitors as the energy storage device, it just takes 0.8 s, 2.2 s, and 4.5 s to charge the capacitors to 3 V under the rotation of 600 r min^−1^, respectively, as shown in Fig. 5f. The rotary DC-TENG with PVC film as the triboelectric layer, the high DC output can directly drive the electronic devices, e.g., a commercial thermo-hygrometer or four commercial LED bulbs, as shown in Fig. 5g, Supplementary Movie 1, 2. The self-powered system consisting of the rotary DC-TENG (the energy supply unit), capacitor (660 μF, the energy storage unit), and commercial thermo-hygrometer (the energy consumption unit) is built, and the corresponding circuit is shown in Supplementary Fig. 21. The monitored voltage of the capacitor at different rotary DC-TENG working conditions is shown in Fig. 5h. When the energy supply by DC-TENG is shut down, the charges in the capacitor are gradually consumed by the thermo-hygrometer, resulting in the decrease of the voltage of the capacitor. With the extra energy supplied by the DC-TENG, the voltage starts to increase because the supplied energy is larger than that consumed by thermo-hygrometer. However, as the energy supply is shut down again, the voltage of the capacitor falls again due to the consumption of thermo-hygrometer (Fig. 5h).

## Discussion

In summary, we propose a set of selection rules of triboelectric materials in direct current (DC-TENG) based on surface charge density, friction coefficient, polarization, the utilization rate of charges, and stability to screen whether a triboelectric material is suitable to be used in DC-TENG. Taking advantage of this model, the PVC film is found to be utilized as the triboelectric layer for DC-TENG, whose effective charge density can reach 8.80 mC m^−2^, which is >20 times that of the previous DC-TENG, and breaks the existing record of various-type TENGs. Furthermore, the proposed comprehensive model can be used to provide a guideline for the triboelectric material selection of DC-TENG to satisfy its practical application requirements. With the widespread applications of TENG, the database of triboelectric materials used in TENG becomes more abundant. The selection rule is not limited to the matching of copper and organic materials, but can also be used in other suitable combinations of triboelectric materials, such as other metals and organic materials or organic materials and organic materials, to obtain higher DC output. Moreover, on the basis of the selection model, extensive data search and prediction can be carried out with the help of machine learning or big data analysis, so as to realize the intelligent design of triboelectric materials for high-performance DC-TENG.

## Methods

### Triboelectric materials

The manufacturer of the triboelectric materials used in this work is listed in Supplementary Table 2.

### Fabrication of sliding AC-TENG

The sliding AC-TENG device is divided into two parts: the slider with Cu electrode and the stator with triboelectric layer. For the slider, the acrylic board with the thickness of 5 mm is cut into a rectangle with the size of 10 mm × 20 mm as the substrate by a laser cutter machine (PLS6.75, Universal Laser Systems), and a copper foil with the same size of the substrate adheres on the acrylic substrate as the upper electrode. For the stator, the acrylic board is cut into a rectangle with the size of 20 mm × 60 mm, and the same size foam is pasted on the acrylic substrate, then, a piece of conductive fabric with the size of 20 mm × 30 mm is stick on one side of the surface of the foam as the bottom electrode. The different triboelectric materials adhere to the bottom electrode as the different triboelectric layers.

### Fabrication of sliding microstructure-designed DC-TENG

The acrylic board is cut into a rectangle shape with the size of 10 mm × 30 mm × 3 mm as the slider, and then the interlaced grooves with different depths are carved on the slider. Taking the microstructure-designed DC-TENG with 20 DC units as an example, the number of deep grooves is 20 and the number of shallow grooves is 21. The copper wire (*Φ* = 250 µm) is embedded into deep grooves as the FEs, and stainless steel wire (*Φ* = 100 µm) is embedded into shallow grooves as the CCEs. The distance between the centers of FE and CCE is 500 μm. The FE is polished to generate an appropriate distance difference between FE’s surface and CCE’s surface. The acrylic sheet with a thickness of 5 mm is cut into a rectangle substrate with the size of 20 mm × 100 mm as the stator, and the same size foam is adhered to the substrate to achieve soft contact. The different triboelectric materials are pasted on the foam as the triboelectric layers for microstructure-designed DC-TENG. The microstructure-designed DC-TENG devices with different numbers of DC units are prepared with the same method.

### Fabrication of rotary microstructure-designed DC-TENG

According to the photographs of rotary microstructure-designed DC-TENG in Fig. 5d, the stator contains three acrylic cycles, whose outer diameter is 90 mm (inner diameter is 70 mm), 65 mm (inner diameter is 47 mm), and 42 mm (inner diameter is 26 mm), respectively. The preparation method is the same as the sliding rotary microstructure-designed DC-TENG, and controls the narrowest distance between the centers of FE and CCE is ~500 μm (namely the position at the inner ring). The rotator is a circular acrylic substrate covered by the foam and the different triboelectric materials are stick on the foam.

### COMSOL simulation

The 2D electric potential distribution in the gap between CCE and triboelectric layer is calculated with the software COMSOL. The gap distance is set as 35 μm. CCE is stainless steel. The triboelectric materials and corresponding surface charge density are set based on their sliding AC output charge density.

### Characterization and electrical measurement

The programmable electrometer (6514, Keithley Instruments model) was utilized to test the short circuit current and output charge density of various-type TENG. The voltage of the capacitor is monitored by the potentiostat (VSP-300, Bio-logic) during the charging process of the capacitor and the charging/discharging process of the self-charging power system. The sliding and rotary movement processes are achieved by a linear motor (TSMV120-1S, LinMot) and rotary motor (BXS6400CM-A, Orientalmotor), respectively. The micrographs of friction surface and microstructure of DC-TENG are measured by scanning electron microscopy (S4800, Hitachi). The surface roughness value *Ra* was tested by the Surface roughness measuring instrument (TR210, JIMTEC). The ferroelectric tester unit (TF Analyzer 1000, aixACCT) is used to obtain the polarization vs. electric field loops of different triboelectric materials.

## Supplementary information

Supplementary Information

Description of Additional Supplementary Files

Supplementary Movie 1

Supplementary Movie 2

## Data Availability

The data that support the findings of this study are available from the corresponding author upon reasonable request.
