# Supplementary material for: Selection rules of triboelectric materials for direct-current triboelectric nanogenerator
Source: Nat Commun. 2021 Aug 3;12:4686. doi: 10.1038/s41467-021-25046-z (PMC8333059; doi:10.1038/s41467-021-25046-z)
Supplement: Supplementary file 1 — Supplementary Information [file 41467_2021_25046_MOESM1_ESM.pdf]

Supplementary Materials

Selection rules of triboelectric materials for direct-current triboelectric nanogenerator

Zhihao Zhao[1,2,δ], Linglin Zhou[1,3,δ], Shaoxin Li[1,3,δ], Di Liu[1,3], Yanhong Li[1], Yikui Gao[1], Yuebo Liu[1], Yejing Dai[2,*], Jie Wang[1,3,*] and Zhong Lin Wang[1,3,4,*]

[1]Beijing Institute of Nanoenergy and Nanosystems, Chinese Academy of Sciences, Beijing 100083, P. R. China

[2]School of Materials, Sun Yat-sen University, Guangzhou 510275, P. R. China

[3]School of Nanoscience and Technology, University of Chinese Academy of Sciences, Beijing 100049, P. R. China

[4]School of Materials Science and Engineering, Georgia Institute of Technology, Atlanta, GA 30332, USA

[δ]Z. Zhao, L. Zhou and S. Li contributed equally to this work.

[*]Corresponding Author: Y. Dai: daiyj8@mail.sysu.edu.cn; J. Wang: wangjie@binn.cas.cn; Z. L. Wang: zhong.wang@mse.gatech.edu

Content

Supplementary Figures:

Supplementary Fig. 1. Test diagram of the coefficients of friction.

Supplementary Fig. 2. The average friction coefficient of triboelectric materials.

Supplementary Fig. 3. The surface roughness Ra of triboelectric materials.

Supplementary Fig. 4. Schematic diagram of sliding AC-TENG.

Supplementary Fig. 5. The output performance of sliding AC-TENG with different triboelectric materials.

Supplementary Fig. 6. The electric field distribution of various charged friction film and charge collecting electrode.

Supplementary Fig. 7. The electric field distribution of the charged friction film and charge collecting electrode.

Supplementary Fig. 8. Schematic diagram of the influence of polarization intensity on the DC output.

Supplementary Fig. 9. The polarization vs. electric field loops of different triboelectric materials under 10 kV m^{-1}.

Supplementary Fig. 10. The polarization vs. electric field loops for different triboelectric materials under various electric field.

Supplementary Fig. 11. The structure of microstructure-designed DC-TENG.

Supplementary Fig. 12. Friction coefficients between micro-structured copper electrode device and various triboelectric materials.

Supplementary Fig. 13. Schematic diagram of microstructure-designed DC-TENG.

Supplementary Fig. 14. The output of microstructure-designed DC-TENG with different triboelectric materials.

Supplementary Fig. 15. Schematic diagram of DC-TENG with PA as triboelectric material.

Supplementary Fig. 16. The ratio of collected charges with charges formed by contact electrification in one CCE.

Supplementary Fig. 17. The stability of different triboelectric materials for the rotary DC-TENG.

Supplementary Fig. 18. The SEM images before and after the stability test of different triboelectric materials for the rotary DC-TENG.

Supplementary Fig. 19. Schematic diagram of stability degradation by using FEP film as triboelectric material during the DC test.

Supplementary Fig. 20. The output performance of microstructure-designed DC-TENG with different number of DC-TENG units.

Supplementary Fig. 21. The circuit of MDC-TENG directly driving electronic devices with energy storage units.

Supplementary Table:

Supplementary Table 1. The origin and normalized indexes of radar chart.

Supplementary Table 2. The manufacturer of the different triboelectric materials.

Supplementary Notes:

Supplementary Note 1: Working mechanism of DC-TENG.

Supplementary Note 2: Working mechanism of sliding AC-TENG.

Supplementary Note 3: The calculation of coefficient of friction.

Supplementary Note 4: Working mechanism of microstructure-designed DC-TENG.

Supplementary Note 5: Working mechanism of DC-TENG with PA as triboelectric material.

Supplementary Note 6: The stability of PVC, FEP, PTFE, PEEK, PI, and PVDF.

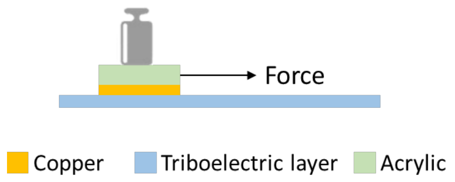

Supplementary Fig. 1. Test diagram of the coefficients of friction.

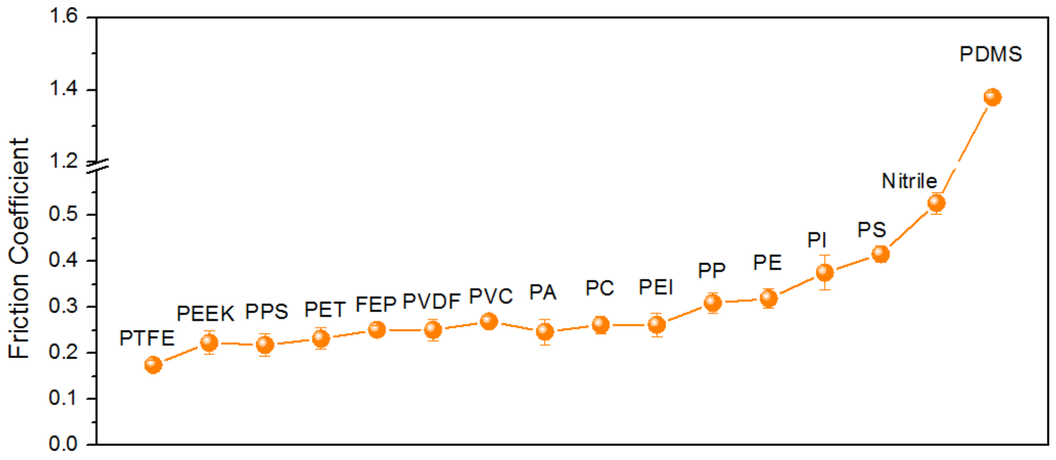

Supplementary Fig. 2. The average friction coefficient of triboelectric materials (error bars represent standard deviation).

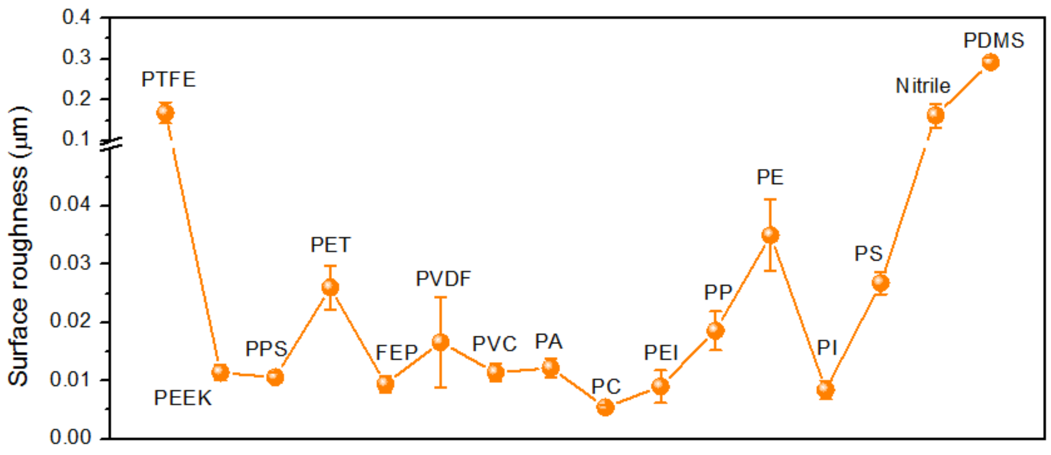

Supplementary Fig. 3. The *Ra* values of surface roughness for different triboelectric materials (error bars represent standard deviation).

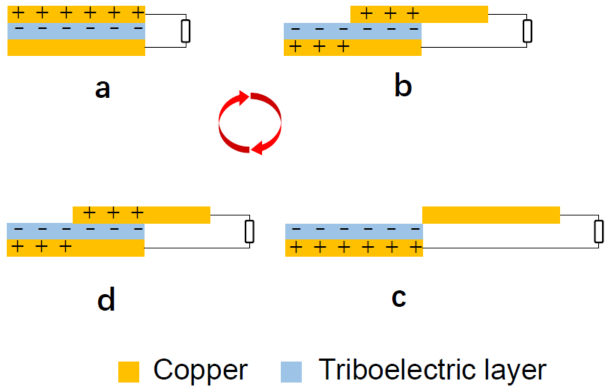

Supplementary Fig. 4. Schematic diagram of sliding AC-TENG at different movement conditions: (a) The initial state, (b) friction electrode moving forward, (c) the end state (d) friction electrode moving backward.

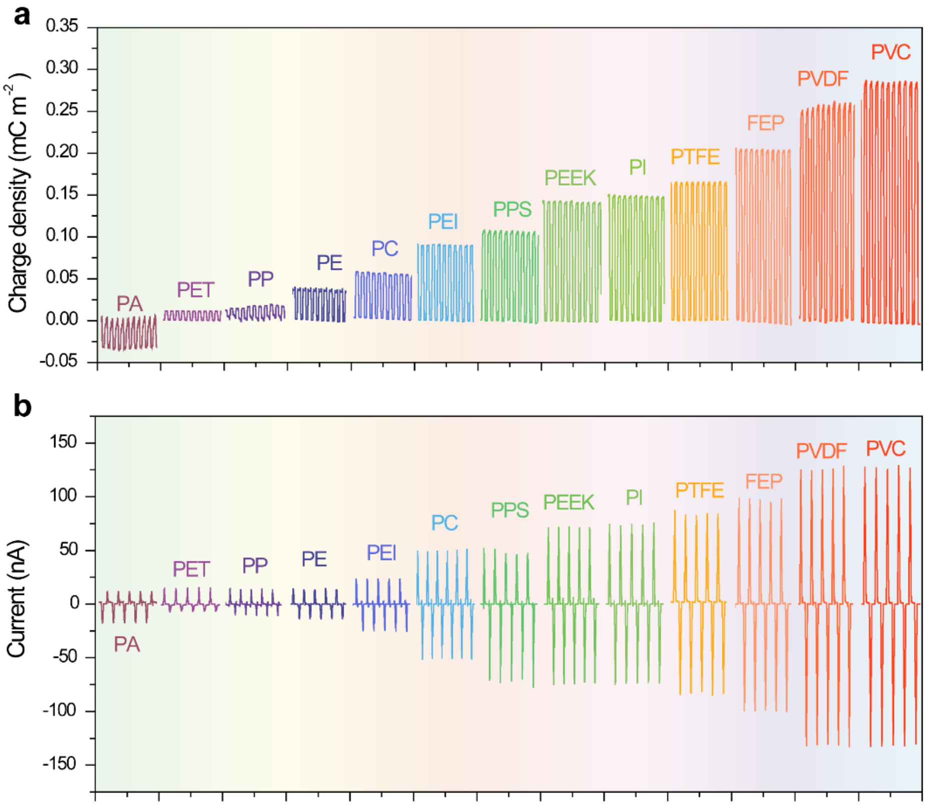

Supplementary Fig. 5. The surface charge density of different triboelectric materials. (a) Charge density and (b) short current of sliding AC-TENG with different triboelectric materials.

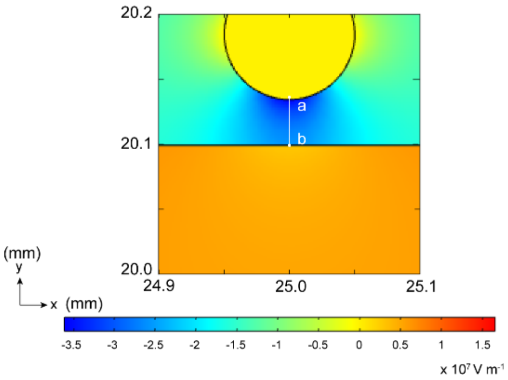

Supplementary Fig. 6. The electric field distribution of the charged friction film and charge collecting electrode.

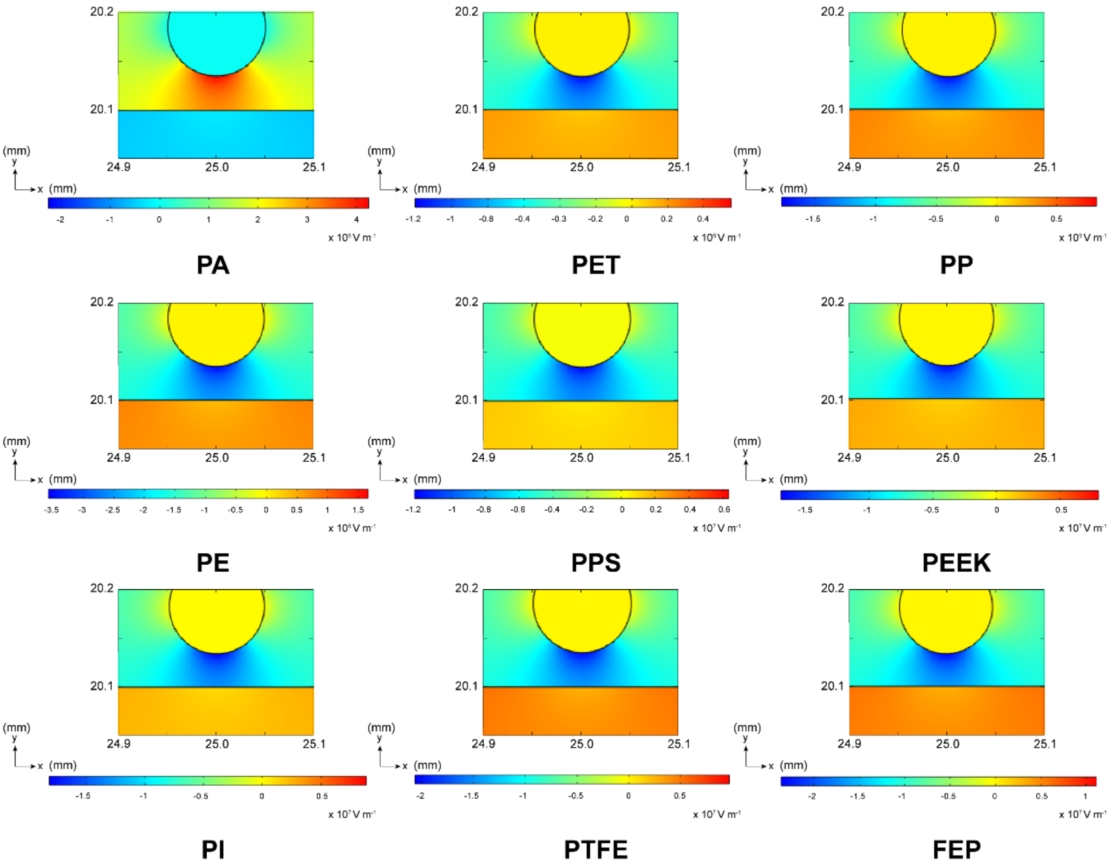

Supplementary Fig. 7. The electric field distribution of various charged friction film and charge collecting electrode.

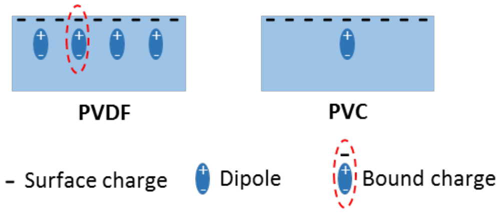

Supplementary Fig. 8. Schematic diagram of the polarization effect influence on the DC output.

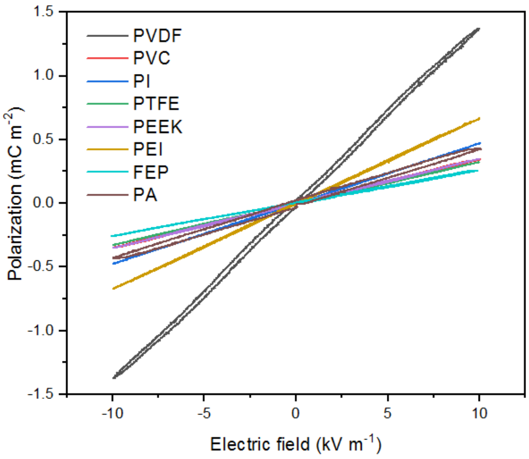

Supplementary Fig. 9. The polarization *vs.* electric field loops of different triboelectric materials under 10 kV m^{-1}.

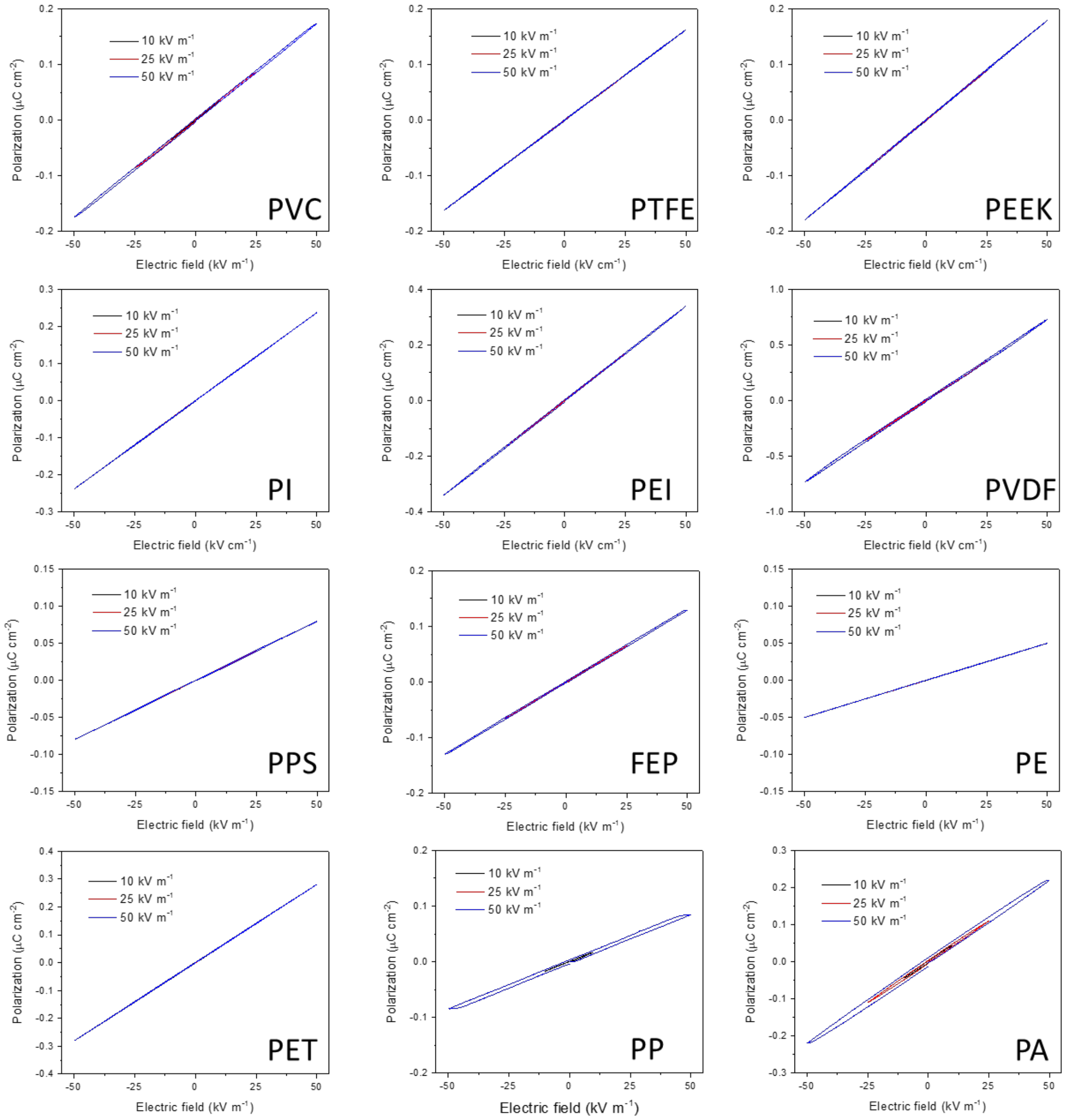

Supplementary Fig. 10. The polarization *vs.* electric field loops for different triboelectric materials under various electric fields.

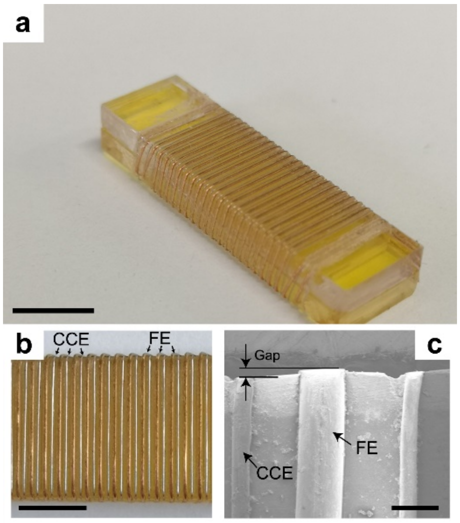

Supplementary Fig. 11. The structure of microstructure-designed DC-TENG. (a) Photograph (scale bar = 1 cm) and (b) enlarge photograph (scale bar = 500 μm) of DC-TENG device with 20 DC units. (c) The scanning electron microscope of DC-TENG device (scale bar = 250 μm).

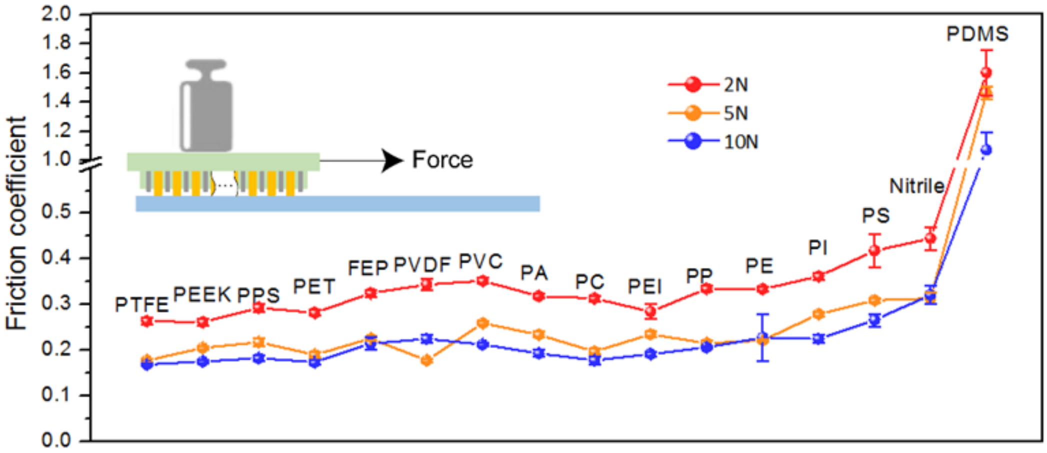

Supplementary Fig. 12. Friction coefficients between micro-structured copper electrode device and various triboelectric materials (error bars represent standard deviation).

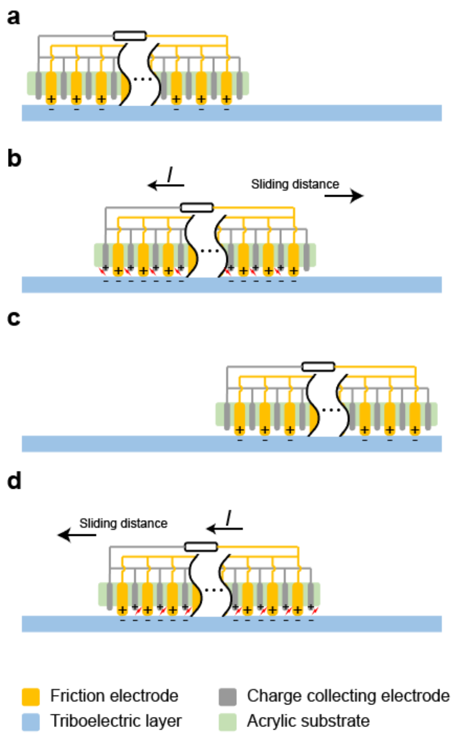

Supplementary Fig. 13. Schematic diagram of microstructure-designed DC-TENG at different movement conditions: (a) The initial state, (b) moving forward, (c) the end state (d) moving backward.

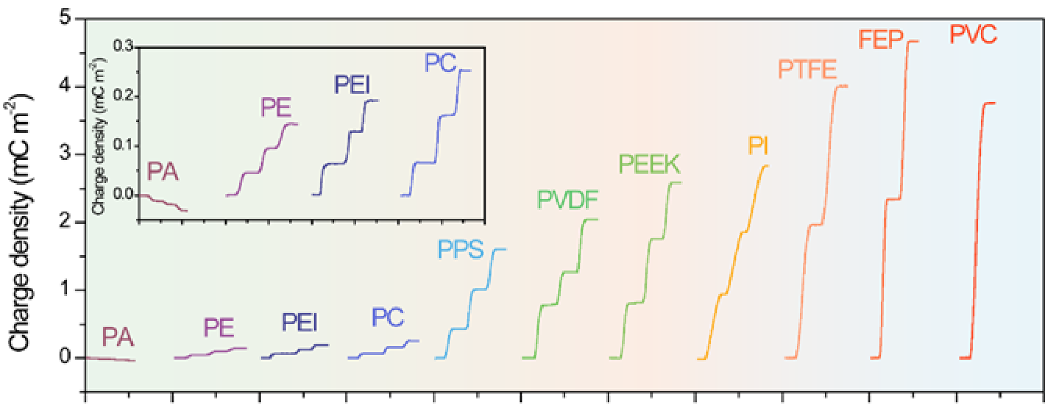

Supplementary Fig. 14. The charge density of microstructure-designed DC-TENG (DC unit: 20) with different triboelectric materials.

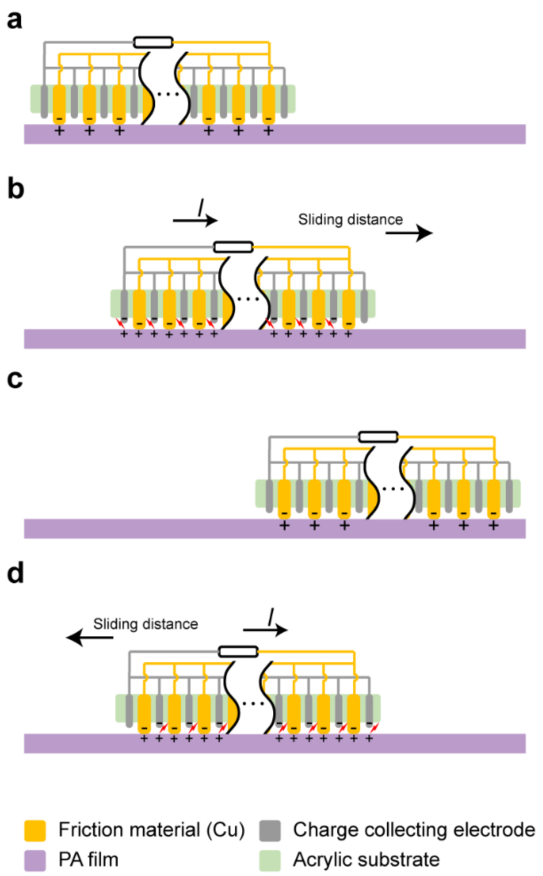

Supplementary Fig. 15. Schematic diagram of DC-TENG with PA as the triboelectric material at different movement conditions: (a) the initial state, (b) moving forward, (c) the end state (d) moving backward.

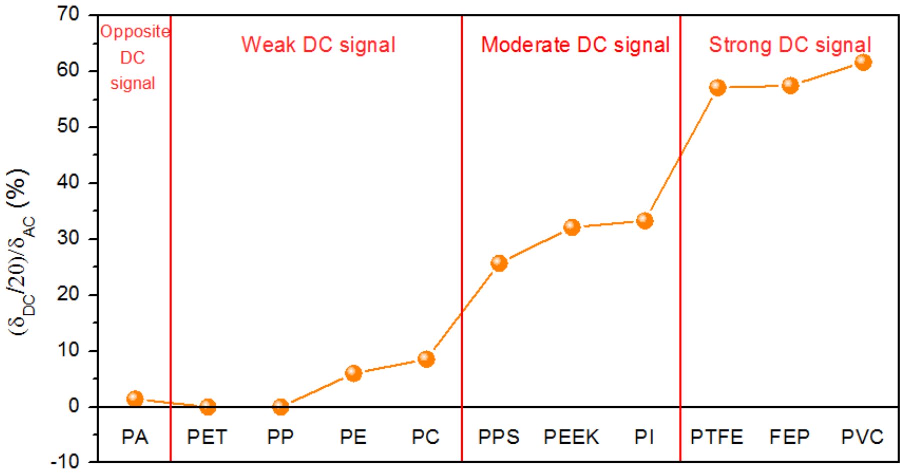

Supplementary Fig. 16. The ratio of collected charges with charges formed by contact electrification in one CCE.

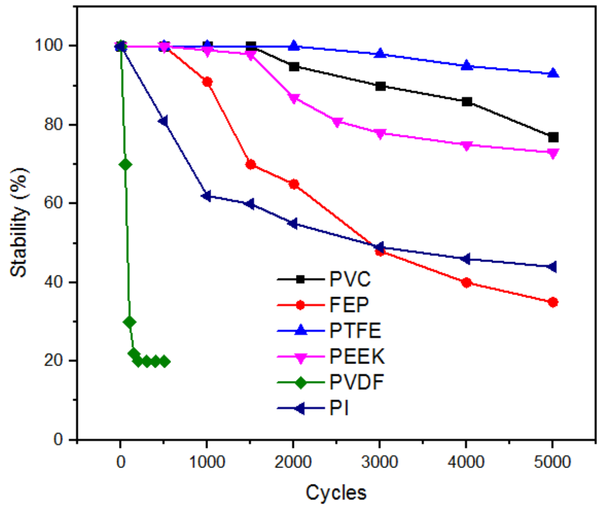

Supplementary Fig. 17. The stability of different triboelectric materials under the rotary DC-TENG.

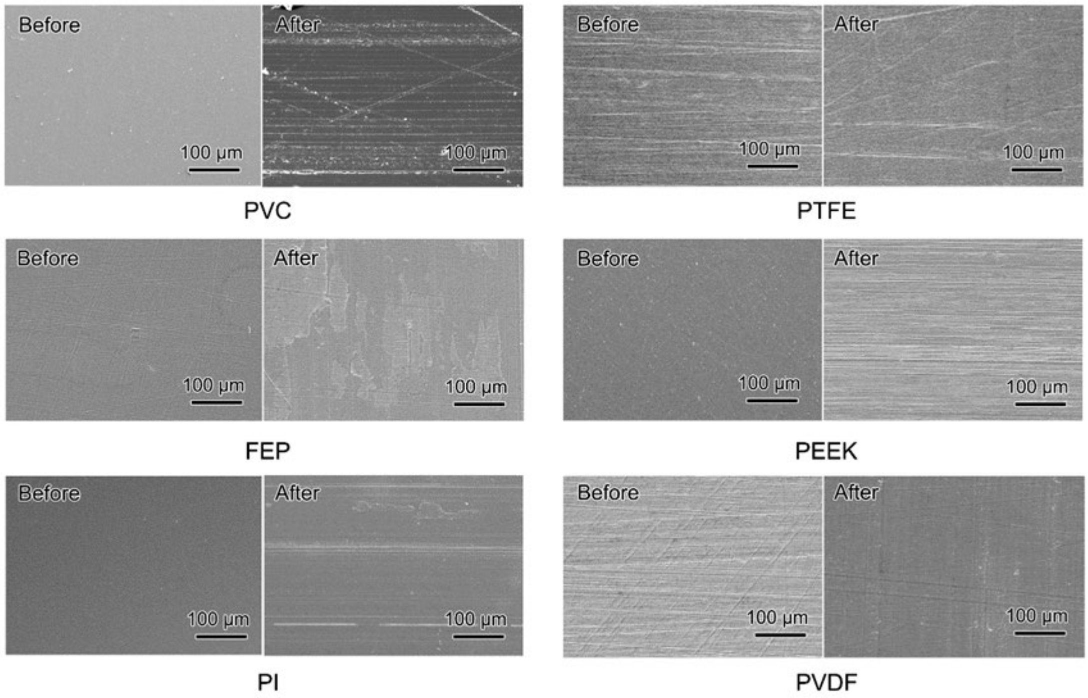

Supplementary Fig. 18. The SEM images of different triboelectric materials before and after the stability test under the rotary DC-TENG.

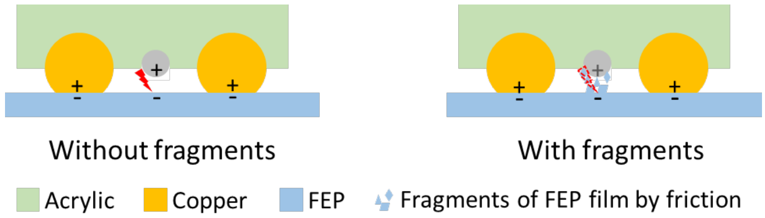

Supplementary Fig. 19. Schematic diagram of stability degradation by using FEP film as the triboelectric material during the DC test.

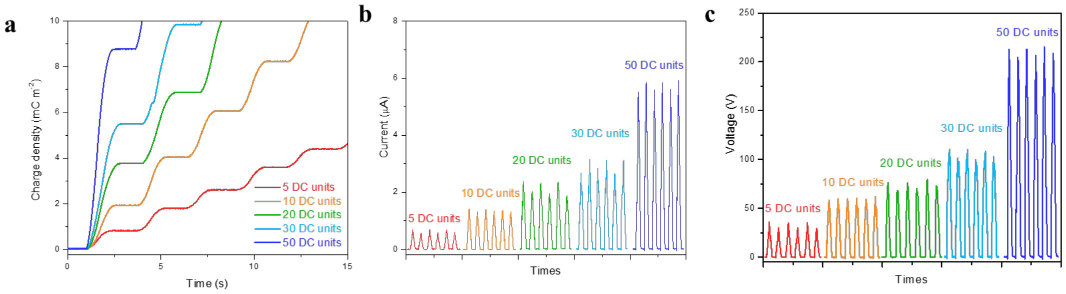

Supplementary Fig. 20. Output performance of microstructure-designed DC-TENG with different number of DC units, (**a**) Output charge density, (**b**) short circuit current and (**c**) open circuit voltage.

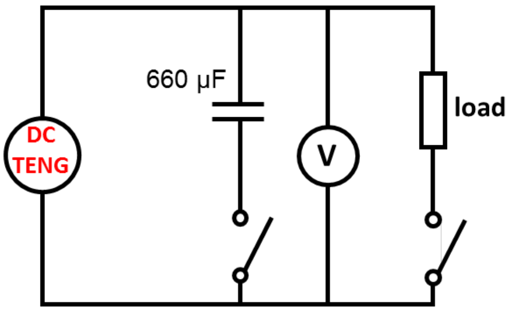

Supplementary Fig. 21. The circuit of MDC-TENG directly driving electronic devices with energy storage units.

Supplementary Table 1. The origin and normalized indexes of radar chart.

		PVC	PTFE	FEP	PEEK	PI	PVDF
Origin indexes	σ_{SCD} (mC m^{-2})	0.30	0.17	0.20	0.14	0.15	0.26
	σ_{DC} (mC m^{-2})	3.70	2.00	2.30	0.90	1.00	0.64
	η (%)	62	58	57	32	33	12
	$1/\mu$	3.7	5.88	4.0	4.3	3.1	3.8
	ρ (%)	77	93	35	78	44	21
Normalized indexes	σ_{SCD}*	1.000	0.567	0.667	0.467	0.500	0.867
	σ_{DC}*	1.000	0.541	0.622	0.243	0.270	0.173
	η*	1.000	0.935	0.919	0.516	0.532	0.194
	$1/\mu$*	0.629	1.000	0.680	0.731	0.527	0.646
	ρ*	0.828	1.000	0.376	0.838	0.473	0.226

Supplementary Table 2. The manufacturer of the different triboelectric materials.

Materials	Manufacturer	Country
PA	Kunshan Yuncheng Plastic Industry Co., Ltd	China
PET	Dongguan Yanming Plastic Trade Co., Ltd.	China
PP	Kunshan Huiliteng Plastic Flim Co., Ltd	China
PE	Kunshan Huiliteng Plastic Flim Co., Ltd	China
PC	Shanghai Xingmei Material Technology Co., Ltd	China
PS	Zhonghao Chenguang Research Institute of Chemical Industry Co., Ltd.	China
PVDF	Nanjing June Hom New Materials Co., Ltd.	China
PEI	GEHR	Germany
PPS	GEHR	Germany
PEEK	GEHR	Germany
PI	Shenzhen Shunyu Adhesive Products Technology Co., Ltd.	China
PTFE	Vendor: Grainger, Catalog No.: 30FZ59 10	USA
FEP	DuPont	USA
PVC	Dongguan Sanhe Packaging Materials CO., Ltd	China
PDMS	Dow corning	USA
Nitrile	AMMEX	USA

Supplementary Note 1: Working mechanism of DC-TENG.

The working mechanism of sliding DC-TENG is the coupling between contact electrification and electrostatic breakdown. As shown in Fig. 1a(i), the DC-TENG possesses a sample structure with friction electrode (FE) which is used for contact electrification with friction layer, and charge collecting electrode (CCE) which collects the charges formed by electrostatic breakdown. During the sliding process, the FE contact with the friction layer (taking PTFE as example), resulting in the positive charges on the FE and negative charges on the PTFE due to the contact electrification. Due to the existence of charges on the PTFE, with the slider sliding further, the followed gap between CCE and PTFE will build a strong electric field, which will lead to the air breakdown in the gap and generate direct current in the external circuit if the electric field is strong enough, as shown in Fig. 1a(ii). When the DC-TENG device slides to the end of PTFE film (Fig. 1a(iii)), the air breakdown process will stop because of the termination of contact electrification.

Supplementary Note 2: The calculation of coefficient of friction.

The kinetic coefficient of friction is calculated as follow:

$$\mu = \frac{A}{B}$$

where A is the average force obtained during the slider uniform sliding on the surface of various films, B is the loading force, as shown in the Supplementary Fig. 1. The arithmetic mean and standard deviation of the coefficient of friction are calculated by five observations.

Supplementary Note 3: Working mechanism of sliding AC-TENG.

The fundamental mechanism of sliding AC-TENG is the coupling of contract electrification and electrostatic induction. As shown in Supplementary Fig. 4a, when the sliding Cu electrode contracts with the dielectric film (for example PTFE), the electrons transfer from Cu to PTFE because of contract electrification effect, resulting in the positive charges in the Cu electrode and negative charges on the surface of PTFE. When the upper electrode slides forward, the upper and bottom electrodes are misaligned, and thus the bottom electrode will induce positive charges to balance the potential difference, resulting in electrons flow from bottom electrode to upper electrode in the external circuit (Supplementary Fig. 4b). The electrons keep flowing until the upper electrode completely misaligned with bottom electrode (Supplementary Fig. 4c). As the upper electrode sliding backward, the electrons flow back to bottom electrode through the external circuit (Supplementary Fig. 4b).

Supplementary Note 4: Working mechanism of microstructure-designed DC-TENG

The working mechanism of microstructure-designed DC-TENG is the same as the DC-TENG with sample structure in Fig. 1. As shown in Supplementary Fig. 13a, the microstructure designed DC-TENG possesses a structure that the multiple FEs (material: Copper) and CCEs (material: Stainless steel) are interlaced arrangement on the acrylic substrate, and the width of FE and CCE is 250 μm and 100 μm, respectively. During the sliding process, taking advantages of contact electrification and air breakdown, the microstructure-designed DC-TENG will generate DC signals (Supplementary Fig. 13b, 13c). When the microstructure designed DC-TENG device slides backward (from Supplementary Fig. 13c to Supplementary Fig. 13d), the same contact electrification and air breakdown process occur and form another direct current peak. It is obvious that if the continuous movement of microstructure-designed DC-TENG device in one direction will generate continuous direct current output.

Supplementary Note 5: Working mechanism of DC-TENG with PA as triboelectric material.

The working mechanism of sliding DC-TENG with PA as triboelectric material is also the coupling between contact electrification and electrostatic breakdown. According to the result of Fig. 3a, the PA film is easier to lose electrons than copper when it contact with copper, and thus the PA film will show positive charges and the Cu electrode will show negative charges, as shown in Supplementary Fig. 15a. As a result, when the slider move forward and backward (Supplementary Fig. 15b-d), the direction of the electric field built in the gap between PA and FE is also opposite to that in the gap between FE with the film which possesses negative charges on its surface (e.g., PTFE).

Supplementary Note 6: The stability of PVC, FEP, PTFE, PEEK, PI, and PVDF.

The PVC, FEP, PTFE, PEEK, PI, and PVDF films were utilized as triboelectric materials to obtain their stability with the rotary DC-TENG device (Fig. 5d(i)) under the long-term DC measurement (speed: 120 r min^{-1}), the corresponding results are shown in Supplementary Fig. 17. Except for PVDF, the other films have been tested for over 5000 cycles. The PTFE shows better stability than the others, which can maintain over 90% output performance compared to the first cycle, and PVC can maintain 75%. The output current of FEP and PI gradually decrease with the operating hours increasing, and after 5000 cycles, the rested current output is less than 50%. The PEEK shows a moderate stability compared with other triboelectric materials. The PVDF film shows inferior stability, whose current output dramatically drop to less than 20% at ~200 cycles. Because the inherent feature of sliding friction of DC-TENG, the wear process cannot be ignored. It can been seen that the films possess scratches after long-term working. The scratches of PEEK and PI show clear edge, indicating the abrasive dust is formed during friction process, and the abrasive dust will block the gap and weaken the air breakdown because of the specialty of DC-TENG structure (Supplementary Fig. 19), as well as the surface mass stripping of FEP. However, the PTFE and PVC, especially the PTFE, show the plastic scratch not the abrasive dust. Although the surface wreck of the triboelectric materials will weaken the contact electrification process, the abrasive dust will not significantly affect the air breakdown process. Thus, the PTFE and PVC shows a relatively good stability when utilized as the triboelectric materials for DC-TENG.
