# Supplementary material for: Selection rules of triboelectric materials for direct-current triboelectric nanogenerator
Source: Nat Commun. 2021 Aug 3;12:4686. doi: 10.1038/s41467-021-25046-z (PMC8333059; doi:10.1038/s41467-021-25046-z)
Supplement: Supplementary file 2 — Description of Additional Supplementary Files [file 41467_2021_25046_MOESM2_ESM.docx]

**Description of Additional Supplementary Files**

**Supplementary Movie 1:** Commercial LED bulbs are driven directly by rotary DC-TENG with PVC as triboelectric material.

**Supplementary Movie 2:** Commercial thermo-hygrometer is driven directly by rotary DC-TENG with PVC as triboelectric material.
